# Exercise Intervention in PTSD: A Narrative Review and Rationale for Implementation

**DOI:** 10.3389/fpsyt.2019.00133

**Published:** 2019-03-21

**Authors:** Nicole J. Hegberg, Jasmeet P. Hayes, Scott M. Hayes

**Affiliations:** ^1^VA Boston Healthcare System, Memory Disorders Research Center, Boston University School of Medicine, Boston, MA, United States; ^2^Department of Psychology, The Ohio State University, Columbus, OH, United States; ^3^Chronic Brain Injury Initiative, The Ohio State University, Columbus, OH, United States

**Keywords:** aerobic exercise, physical activity, fitness, emotion regulation, cognition, MRI, fMRI, PTSD

## Abstract

Posttraumatic stress disorder (PTSD) is a prominent mental health problem in veteran and community populations. There is accumulating evidence to suggest that aerobic exercise may serve as an effective treatment option for individuals with PTSD. The purpose of this review is to summarize the existing literature exploring aerobic exercise and PTSD and briefly discuss potential mechanisms of PTSD symptom reduction. A search of electronic databases and reference sections of relevant articles published through October 1, 2018 revealed 19 relevant studies that examined aerobic exercise and PTSD symptomatology. A narrative review of extant studies provides encouraging evidence that aerobic exercise interventions alone or as an adjunct to standard treatment may positively impact PTSD symptoms. Potential mechanisms by which aerobic exercise could exert a positive impact in PTSD include exposure and desensitization to internal arousal cues, enhanced cognitive function, exercise-induced neuroplasticity, normalization of hypothalamic pituitary axis (HPA) function, and reductions in inflammatory markers. Randomized clinical trials and translational neuroscience approaches are required to clarify the efficacy of exercise intervention for PTSD and elucidate potential mechanisms of exercise-induced PTSD symptom reduction.

## Introduction

Posttraumatic stress disorder (PTSD) can develop after exposure to life-threatening and highly distressing events such as military combat, accidents, assault, or natural, or human-caused disasters. PTSD is a prominent mental health problem in veteran and community populations, affecting 7.6% of OEF/OIF veterans (1) and 3.5% of adult Americans (2). According to the DSM-5, the diagnosis of PTSD is characterized by four broad symptom clusters that include intense reliving of the traumatic event through disruptive memories and nightmares, avoidance of reminders of the event, negative cognitions and mood, and hyperarousal. The impact of PTSD is multi-faceted. In addition to the characteristic symptoms of PTSD, impaired cognitive performance (3, 4) and alterations in brain structure and function (5, 6) are well-documented. Further, individuals with PTSD have increased rates of health-care utilization, loss of productivity, and major chronic diseases (7, 8). For instance, individuals with PTSD have higher rates of diabetes, obesity, and metabolic syndrome (9–12). Moreover, individuals with PTSD exhibit reduced participation in regular physical activity relative to pre-PTSD time periods (13), which likely contributes to the medical, cognitive, and neural comorbidities associated with sedentary behavior (14–16).

Although there are evidence-based psychological and pharmacological (17) treatments that have proven to be effective in treating the characteristic symptoms of PTSD, there are often barriers to treatment initiation among those suffering from PTSD, including stigma, motivation, cost, and access to care (18). While exercise is not without its barriers, such as limited motivation, self-efficacy, or time, it is one intervention that is broadly accessible, low-cost, and could avoid the negative connotations associated with traditional mental health treatment approaches (19). Given the constellation of psychiatric, cognitive, and general health issues associated with PTSD, it is important to consider the case for aerobic exercise for the treatment of PTSD, which may have the potential to enhance functioning in each of these areas. Indeed, aerobic exercise has the potential to exert a positive impact on PTSD via both psychological and neurophysiological mechanisms, such as exposure and desensitization to internal arousal cues, enhanced cognitive function, exercise-induced neuroplasticity, normalization of hypothalamic pituitary axis (HPA) function, and reductions in inflammatory markers.

The notion that physical activity and exercise are viable treatments for PTSD has been gaining momentum in the research community (20–25). The terms are often used interchangeably but are distinct from one another. Physical activity is “any bodily movement produced by skeletal muscles that results in energy expenditure” [(26), p 126]. Physical activity can be in the form of walking, cycling, sport, household or occupational activities, and can vary widely in the amount of energy expenditure (see Table 1 for description of exercise intensities). Regular physical activity often results in improved balance, coordination, musculoskeletal strength, and/or aerobic fitness, depending on the type. The term exercise is commonly used in the literature and refers to a subcategory of physical activity that is planned, structured, repetitive, and intended to improve or maintain physical fitness (26). Cardiorespiratory fitness is one type of physical fitness that refers to the ability to perform large-muscle, dynamic, moderate to high-intensity physical activity for a prolonged period, and depends on the cardiovascular, respiratory, and skeletal muscle systems. Regular physical activity and/or exercise that is tailored to improve cardiorespiratory fitness, such as walking, dancing, or cycling, has significant health benefits such as reducing the risk of cardiovascular disease, diabetes, and obesity (28). Benefits of regular physical activity and/or exercise also extend to mental health (29–31). In fact, research suggests aerobic exercise, which improves cardiorespiratory fitness, is an effective treatment for depression, anxiety, and schizophrenia (32–36) through both physiological and psychological mechanisms, and may be comparable or superior to other common treatments, such as psychotherapy and pharmacology (37–39). Although PTSD shares some symptomatic overlap with depression and anxiety disorders, there is consensus that PTSD is a distinct disorder, in terms of etiology, symptomatology, and neurobiological correlates. Treatments that are highly efficacious for the treatment of depression may be less effective in PTSD treatment. Therefore, the purpose of this review is to provide a summary of the extant literature examining the impact of exercise on PTSD specifically. We discuss possible mechanisms underlying the potential link between aerobic exercise and PTSD symptoms, and conclude with future directions for aerobic exercise studies in PTSD.

**Table 1:** Exercise intensities.

**Intensity**	Percentage of Maximal HR^*^	Oxygen Consumption^*^	**Examples**
Light	57–63%	< 3 METs	Slow walk, household chores
Moderate	64–76%	3–6 METs	Brisk walk, slow jog, easy bike riding, tennis
Vigorous	77–95%	>6 METs	Running, hard bike riding, fast swimming, basketball, soccer

* *Values from Garber et al. (27). HR, heart rate; MET, metabolic equivalent*.

## Methods

For this narrative review, we focused on aerobic exercise studies targeting enhancement of cardiorespiratory fitness. To identify studies examining the impact of aerobic exercise on PTSD symptoms, we completed a search of electronic databases (PubMed, Google Scholar, PsycINFO) for relevant studies published through October 1, 2018 using the search terms “PTSD” and “exercise' or “physical activity” or “walking” or “cycling” or “high intensity interval training” or “aerobic fitness” or “cardiorespiratory fitness.” We also searched the reference section of relevant articles. To be included in the review, studies had to include an assessment of PTSD symptomatology (in trauma-exposed community or clinical samples) and metrics of aerobic physical activity or exercise or implement an aerobically based exercise intervention in patients with PTSD (or assess PTSD symptoms). If the intervention was combined with an aerobic component (e.g., aerobic + resistance training), we included it in our review. We excluded other exercise interventions that have been explored in the context of PTSD, such as yoga (40), which typically elicits smaller changes in cardiorespiratory fitness than aerobic-focused exercise programs or have a dearth of studies to draw upon for review (e.g., resistance training alone). Our search revealed 19 relevant studies (nine observational and 10 intervention) that examine aerobic exercise and PTSD symptomatology in community and clinical samples of trauma-exposed individuals with methodology exploring correlational, preventative, and treatment effects.

## Results and Discussion

### Observational Research

An aggregate of correlational studies suggests that exercise reduces PTSD symptoms (see Table 2). Of note, the majority of studies assessed PTSD using DSM-IV criteria or using PTSD questionnaires that assess the three broad symptom clusters defined in the DSM-IV (re-experiencing, avoidance/numbing, and hyperarousal). In one prospective study of 38,883 randomly selected U.S. veterans (77% male), 96% of which did not have PTSD at baseline, participants who reported engaging in vigorous exercise (≥ 20 min, two or more times per week), but not combined light or moderate intensity, had significantly decreased odds of developing new PTSD symptoms (odds ratio [OR] 50.58, 95% confidence interval [CI] 0.49, 0.70) or having persistent symptoms (OR 50.59, 95% CI 0.42, 0.83) at 3–5 year follow-up (46). Similarly, Harte et al. (45) found in a cross-sectional analysis of community recruited trauma-exposed adults (*N* = 108; mean age = 23.9; 58% male; 81.7% white) that self-reported vigorous-intensity exercise, but not light- or moderate-intensity exercise, was significantly and inversely associated with PTSD hyperarousal symptom severity on a self-report questionnaire (Posttraumatic Diagnostic Scale [PDS]; β = −0.22, *p* = 0.04). One caveat was that the participants in the study endorsed relatively low levels of PTSD symptomatology (PDS total score: *M* = 7.6, *SD* = 8.75) and did not suffer from other psychopathology, suggesting the findings may not be reflective of associations in clinical samples with greater severity of PTSD or other co-morbid conditions.

**Table 2:** Observational studies of exercise and PTSD.

**Study**	***N***	**Age**	**Gender**	**Ethnicity**	**Population/ Dx criteria**	**Study Design**	**EX Type/ Measure**	**Outcome Measure**	**Results**	**Significance**
Arnson et al. (41)	55	Mean: 49.7 ± 7.5 Range: 19–60	0% female	NR	Israeli veterans/DSM-IV PTSD	CS	Self-reported EX/(single item: regular, infrequent, or none)	CAPS	Severity of PTSD did not differ across EX groups	*p* = 0.52
Bosch et al. (42)	76	Mean: 36.4 ± 9.9 Range: NR	18% female	58% white	OEF/OIF/OND veterans/ DSM-IV-TR full or subthreshold PTSD	Longitudinal	Self-reported EX/(dichotomous variable: engaging in exercise or not)	PCL-M 1 year FU	Baseline EX not significantly related to PTSD sxs at FU	*p* = 0.57
Bourn et al. (43)	239	Mean: 50 ± 15.3 Range: 21–83	9% female	88% white	U.S. Veterans/ CAPS full or subthreshold PTSD	CS	Self-reported EX/GLTEQ	CAPS	Among those reporting high levels of pain severity and interference, individuals who reported being active reported fewer PTSD sxs than those who reported being inactive	^*^
Davidson et al. (44)	346	Mean: 45.5 ± 14.27 Range: NR	19% female	60% white	U.S. veterans/residential treatment for PTSD	CS	Self-reported EX/(single item: days of EX per week)	PCL-M	EX not correlated with PTSD sxs	n.s.
Harte et al. (45)	108	Mean: 23.9 ± 10.22 Range: 18–62	42% female	92% white	Trauma-exposed community/DSM-IV Criterion A	CS	Self-reported EX/ EHQ-R	PDS	Vigorous, but not light- or moderate-intensity EX, was significantly and inversely related to hyperarousal PTSD sxs	^*^
LeardMann et al. (46)	38,883	Mean: NR Range: NR	23% female	83% white	U.S. Veterans/PCL-C	Longitudinal	Self-reported EX/ 2001 National Health Interview Survey	PCL-C	Engaging in vigorous EX (≥20 min, 2 + times/wk) resulted in: Decreased odds of developing sxs; Decreased odds of having persistent PTSD sxs at follow up	OR = 0.58 [0.049–0.70] OR = 0.59 [0.42–0.83]
Rosenbaum et al. (47)	76	Mean: 47.6 ± 11.9 Range: NR	17% female	NR	Inpatient/DSM-IV PTSD	CS	Self-reported EX/ IPAQ-SF	PCL-C	Total walking time negatively associated with PTSD sxs; moderate and vigorous EX not significantly correlated with PTSD sxs	^***^ n.s.
Vujanovic et al. (48)	86	Mean: 24.3 ± 10.54 Range: NR	58% female	89.5% white	Trauma-exposed community/DSM-IV Criterion A	CS	Self-reported EX/ EHQ-R	PDS	Regular smokers who also endorsed a high level of weekly EX had fewer PTSD sxs than low-level exercisers	^*^
Whitworth et al. (49)	182	Mean: 34.6 ± 13.3 Range: 18–69	72% female; 1% other	63.2% white	Adults/PTSD per PCL-C	Longitudinal	Self-reported EX/ GLTEQ	PCL-C 3 month FU	Total EX: - Directly related to avoid/numbing at FU Indirectly associated with total PTSD sxs through alcohol use at FU Strenuous EX: -Directly related to avoid/numbing and hyperarousal sxs at FU - Indirectly related to total PTSD, avoid/numbing, and hyperarousal sxs through sleep at FU	^*^ 95% CI [0.18–1.42] ^*^ 95% CI [−2.43 to −0.05] 95% CI [−1.88 to −0.07]

*CAPS, Clinician-Administered PTSD Scale; CS, cross-sectional; Dx, diagnostic; EHQ-R, Exercise Habits Questionnaire- Revised; EX, exercise; FU, follow-up; GLTEQ, Godin Leisure-Time Exercise Questionnaire; IPAQ-SF, International Physical Activity Questionnaire- Short Form; NA, not applicable; NR, not reported; n.s., not significant; PCL-C, Posttraumatic Stress Disorder Checklist–Civilian Version; PCL-M, Posttraumatic Stress Disorder Checklist–Military Version; PDS, Posttraumatic Diagnostic Scale; sxs, symptoms; +, sample deemed too small to conduct traditional significance tests. No observational studies assessed cardiorespiratory fitness*.

* p < 0.05;

*** *p < 0.001*.

The link between vigorous-intensity exercise and PTSD was further supported by Whitworth et al. (49) who longitudinally assessed 182 community-recruited adults with PTSD (mean age: 34.6 ± 13.3, 72% female, 63% white) at baseline and 3 month follow-up. Findings suggested that those who reported engaging in vigorous-intensity exercise (vigorous running or cycling) but not moderate intensity exercise (non-exhaustive sports) on the Godin Leisure-Time Questionnaire (GLTEQ) had fewer avoidance/numbing (β = −2.18, *p* = 0.05) and hyperarousal (β = −1.87, *p* = 0.03) symptoms at follow-up, assessed with the PTSD Checklist-Civilian screening (PCL-C). For those participants reporting both moderate- and vigorous-intensity exercise, the longitudinal association between total exercise and PTSD was observed only for avoidance/numbing symptoms (β = −1.76, *p* = 0.05). Moderate-intensity activity alone was not associated with total PTSD symptoms or any of the symptom clusters at follow-up. Taken together, the findings suggest that those with low levels of PTSD who engage in vigorous-intensity activity may see the most profound effects on hyperarousal symptoms, whereas there may be more widespread symptom effects as PTSD severity increases.

In contrast to the evidence supporting the link between vigorous-intensity exercise and PTSD, a cross-sectional study found that light-intensity exercise (time spent walking) endorsed on a self-report questionnaire (International Physical Activity Questionnaire- Short Form; IPAQ-SF) was significantly and negatively correlated with PTSD symptoms (β = −0.4; *t* = −3.4; *p* < 0.001) in an inpatient clinical sample (*N* = 76; mean age = 47.6; 83% male), but moderate- and vigorous-intensity exercise were not (47). There are also studies that have failed to establish a significant association between exercise and PTSD symptomatology (41, 42, 44). However, methodological limitations may explain these inconsistencies. For example, studies that failed to detect a significant association measured exercise with a single-item questionnaire or variable (e.g., assessing engagement in exercise or not, or number of days per week of exercise), whereas studies that have found significant effects used validated, multiple-item exercise assessment measures. A single-item measure does not capture frequency, intensity, and time (duration) and type of exercise, and therefore may not be adequate to assess behaviors that promote alterations in cardiorespiratory fitness or the effectiveness of aerobic exercise.

Additional cross-sectional studies highlight co-morbid health concerns as moderators of the association between exercise and PTSD symptoms. For instance, it has been shown that exercise moderates the association between regular smoking and PTSD symptoms in a non-clinical sample of trauma-exposed individuals (*N* = 86; mean age = 24.3; 42.9% male, 89.5% white), such that regular smokers who also endorsed high levels of weekly exercise had fewer hyperarousal *(*β = −0.33, *p* = 0.05), and avoidance (β = −0.38, *p* = 0.04) symptoms of PTSD than their low-level exercise counterparts (48). Moderating effects have also been observed in clinical samples with PTSD. Despite no main effect of exercise on PTSD severity in PTSD treatment-seeking veterans (*N* = 239; mean age = 50; 91.1% male; 87.5% white), among veterans who endorsed high levels of pain severity (β = −0.031, *p* = 0.031) and pain interference (β = −0.142, *p* = 0.025), those who reported being physically active reported fewer PTSD symptoms than those who reported being inactive (43).

The observational research provides promising support for the association between exercise and PTSD along with indications of some variables that could influence this association. These studies suggest that the effect of exercise may vary by disorder severity and/or co-morbid health concerns, which might also serve as a marker for disorder severity. Further, findings suggest that exercise might target different symptoms based on intensity (i.e., vigorous-intensity and hyperarousal symptoms), which may provide insight into potential mechanisms of action. For instance, it may be that vigorous-intensity exercise allows for exposure and desensitization to arousal cues, thereby reducing the heightened response to these cues often seen in hyperarousal. These observational studies, however, are not without their limitations. Many of the studies rely on retrospective self-report measures of exercise, which may be prone to bias, inaccuracies, and misunderstanding of questions [e.g., (50–52)]. Several studies did not report time since the traumatic event, and thus the extent to which chronicity of PTSD symptoms relates to the benefits of exercise is unknown. Moreover, given the cross-sectional nature of many of these studies, the directionality of the relationship between exercise and PTSD symptoms is unknown. That is, an association between exercise and PTSD symptoms might suggest that exercise leads to a reduction in PTSD symptoms, but it does not rule out the possibility that those individuals with lower levels of PTSD tend to exercise more.

### Intervention Research

We identified 10 published studies that explored the impact of exercise interventions on PTSD symptoms, using a randomized clinical trial (RCT), quasi-RCT, or single-group intervention design. These studies suggest that exercise may be effective as a stand-alone treatment or as an augmentation to traditional PTSD treatment (see Table 3).

**Table 3:** Exercise intervention studies and PTSD.

**Study**	***N***	**Age**	**Gender**	**Ethnicity**	**Population/ Dx criteria**	**EX Type/ Measure**	**Fitness indices**	**Control**	**Outcome Measure**	**Results**	***p***
Babson et al. (53)	217	Mean: 52.2 ± 7.06 Range: 24–70	0% female	NR	U.S. veterans/residential treatment for PTSD	Cycling while in group cycling program/cyclometer on road bike; no specifications about frequency, intensity or duration	NA	None	PCL-M	For those reporting poor baseline sleep quality, time spend cycling improved hyperarousal PTSD sxs	^*^
Diaz and Motta (54)	12	Mean: 15.42 ± 0.79 Range: 14–17	100% female	17% white	Residential treatment center/ DSM-IV PTSD	5 week aerobic EX program (25 min moderate walking 60–90% HR at completion, 3x/wk)	NA	None	CPSS; TSCC	Reduced PTSD sxs and trauma severity;1 month follow up mixed for 3 participants remaining at facility	^**^
Fetzner and Asmundson (55)	33	Mean: 36.9 ± 11.2 Range: NR	76% female	79% white	Community sample/ DSM-IV-TR full or subthreshold PTSD	2 week aerobic EX program (20 min EX at 60-80% HR, 6x/intervention) with attentional focus (distraction or somatic sensations)	YMCA ergometer bike test at baseline	EX program without attentional focus	PCL-C	Reduced PTSD symptoms regardless of attentional focus	^**^
Goldstein et al. (56)	47	Mean: 46.8 ± 14.93 Range: 24–69	19% female	40% white	U.S. veterans/DSM-IV full or subthreshold PTSD	12 week EX program (3, 1hr exercise sessions combining aerobic and resistance exercise and yoga movements)	NA	Waitlist	CAPS	Those participating in EX program reported greater reduction in PTSD sx severity compared to waitlist controls	^*^
LeBouthillier et al. (57)	32	Mean: 36.75 ± 11.3 Range: NR	75% female	78% white	Community sample/DSM-IV-TR full or subthreshold PTSD	2 week aerobic EX program (20 min EX at 60–80% HR, 6x/intervention)	Baseline YMCA ergometer bike test (VO_2_ max)	None	PCL-C	Those with lower baseline fitness reported a greater reduction in avoidance and hyperarousal PTSD sxs, compared to those with higher baseline fitness	^**^
Manger and Motta (58)	9	Mean: 48.1 Range: NR	NR	NR	Community sample/CAPS ≥ 20	10 week aerobic EX program (2–3x/wk, 30 min walk or jog at moderate intensity (60–80% max HR), 10 min cool down ≥12x/intervention)	NA	None	PDS	Reduced PTSD sxs after intervention and at 1-month follow-up	^*^
Newman and Motta (59)	11	Mean: 15.7 Range: 14–17	100% female	36% white	Residential treatment center/DSM-IV PTSD	8-week aerobic EX program (40 min with 20 min at 60–80% max HR, 3x/wk)	NA	None	CPTSDI; PTSD-RI	Reduced PTSD sxs compared to baseline;fewer individuals met criteria for PTSD	^***^ ^***^
Powers et al. (60)	9	Mean: 34 ± 11.82	89% female	89% white	Community sample/DSM-IV PTSD	12 session moderate aerobic EX (stationary cycling at 70% age predicted HR) plus PE	NA	PE	PSSI	Greater reduction in PTSD sxs	+ *d* = 2.65
Rosenbaum et al. (61)	81	Mean: 48 ± 12.1 Range: 23–73	16% female	NR	Inpatient population/DSM-IV-TR PTSD	12 week EX program (progressive resistance training 30 min, 3x/wk and walking 10,000 steps/day) + inpatient care as usual	6-min walk test, resting HR, blood pressure	Inpatient care as usual	PCL-C	Greater reduction in PTSD sxs; groups did not differ in fitness level post intervention (no pre- to post-intervention fitness analyses)	^*^
Shivakumar et al. (62)	16	Median: 34	100% female	14% white	U.S. veterans/CAPS ≥ 45	12 week aerobic EX program (brisk walking at 3 mi/hr for 30–40 min, 4x/wk)	NA	None	CAPS; PCL	Improved overall PTSD symptoms	^*^

*CAPS, Clinician-Administered PTSD Scale; CPSS, Child PTSD Symptom Scale; CPTSDI, Child PTSD Inventory; Dx, diagnostic; EHQ-R, Exercise Habits Questionnaire- Revised; EX, exercise; GLTEQ, Godin Leisure-Time Exercise Questionnaire; IPAQ-SF, International Physical Activity Questionnaire- Short Form; NA, not applicable; NR, not reported; n.s., not significant; PCL-C, Posttraumatic Stress Disorder Checklist–Civilian Version; PCL-M, Posttraumatic Stress Disorder Checklist–Military Version; PDS, Posttraumatic Diagnostic Scale; PE, prolonged exposure therapy; PSSI, PTSD Symptom Scale-Interview; PTSD-RI, UCLA PTSD Reaction Index for DSM-IV; sxs, symptoms; TSCC, Trauma Symptom Checklist for Children; +, sample deemed too small to conduct traditional significance tests*.

* p < 0.05;

** p < 0.01;

*** *p < 0.001*.

#### Exercise Only

One of the first studies in this area (58) assessed the impact of a 10 week aerobic exercise program with multiple baselines on PTSD, anxiety, and depression in 9 non-exercising community members who reported severe PTSD symptomatology in response to a trauma occurring at least 3 months prior during a structured interview [Clinician-Administered PTSD Scale [CAPS]: *M* = 63.67 ± 25.95 (63)]. After the intervention, which included at least 12, 30-min moderate intensity (60–80% max HR) exercise sessions over 10 weeks, and at one-month follow up (participants refrained from exercising between intervention and follow up), participants who completed the exercise intervention reported significant reductions in PTSD [*t*_(8)_ = 3.49, *p* = 0.008 and *t*_(8)_ = 3.30, *p* = 0.011, respectively], as well as depression symptomatology [*t*_(8)_ = 4.26, *p* = 0.003 and *t*_(8)_ = 3.05, *p* = 0.016, respectively] and trait anxiety [*t*_(8)_ = 3.07, *p* = 0.002 and *t*_(8)_ = 2.31, *p* = 0.050, respectively]. The researchers followed up this initial study with another multiple baseline intervention in a sample of institutionalized female adolescents (*N* = 11; age: range 14–17 years; 36% white) who met DSM-IV criteria for PTSD based on a clinical administered scale [Children's PTSD Inventory [CPTSDI]; (64)]. Results from the study showed that, compared to baseline, participants who engaged in an 8 week aerobic exercise program (three times per week, 40 min with 20 min at 60–80% maximum heart rate) at an average of 3 years following the experienced trauma reported reduced PTSD symptomatology after the intervention [*t*_(10)_ = 23.56, *p* < 0.001] such that fewer individuals met full criteria for PTSD (59). Further, at 1 month follow-up, PTSD symptomatology increased but did not revert to baseline level, suggesting that reductions may have been at least partly dependent on the exercise intervention. Similarly, another study (54) of 12 institutionalized female adolescents (age range: 14–17 years; 100% female; 17% white) who met criteria for PTSD based on self-report [≥11; Child PTSD Symptom Scale [CPSS]; (65)], with no clear duration since experiencing trauma, showed that a shorter, 5 week aerobic exercise intervention (three times per week, 25 min of moderate-intensity walking: 60–90% maximum heart rate at completion) also resulted in significant reductions in self-reported PTSD symptoms (CPSS) and trauma symptom severity [Trauma Symptom Checklist for Children [TSCC]; (66)]. Only three participants remained at the facility at 1 month follow up and all continued to report significantly less PTSD symptoms and trauma symptom severity compared to baseline.

A more recent study conducted by Shivakumar et al. (62) resulted in similar findings. A small convenience sample of female veterans who reported PTSD symptomatology on the CAPS (≥45; *N* = 16; median age = 34; 14% white, 50% military sexual trauma) and completed a 12 week moderate-intensity exercise program (30–40 min of brisk walking at 3 mph, four times per week) reported significant reductions in overall PTSD symptomatology based on two separate assessments of symptoms (CAPS & PCL; *z* = −2.395, *p* = 0.017 and *F*_(2, 12)_ = 9.42, *p* = 0.03, respectively). In a larger study of male veterans in a 60- to 90-day residential treatment program (*N* = 217; mean age = 52 years), time spent cycling in a non-standardized group bicycling program reduced hyperarousal symptoms at discharge (β = 0.24, *p* = 0.002), but only for those who reported poor baseline sleep quality (53). Findings suggest that only select individuals might benefit from unstandardized exercise with no clear frequency, intensity, or duration. Authors did not report when the interventions occurred in relation to the participants' experienced trauma(s) in either of these two studies.

Studies with larger sample sizes and randomized conditions show further promise for the beneficial effects of aerobic exercise on PTSD. Fetzner and Asmundson (55) examined the influence of aerobic training and attentional focus to somatic sensations on PTSD symptoms in a community sample (*N* = 33; mean age = 37, 75% female). The exercise program consisted of six, 20-min moderate-intensity aerobic exercise sessions (60–80% heart rate) over 2 weeks. It was implemented, on average, 8 years following the participants' experienced trauma with most occurring 1–4 years prior to intervention. The results indicated that the aerobic exercise training, regardless of attentional focus, reduced total self-reported PTSD symptoms on a questionnaire (PCL-C; *p* < 0.01), which was driven by reductions in avoidance symptoms (*p* < 0.01). Another article published from these data (57) suggested that participants with lower fitness at baseline, measured using the YMCA ergometer bike test which assesses submaximal VO_2_ maximum, reported a greater reduction in PTSD symptoms compared to their higher-fit peers (β = 0.10, *p* < 0.10). These results suggest that individuals with low fitness may preferentially benefit from the effects of aerobic exercise on PTSD. Finally, a randomized study conducted in a veteran population (*N* = 47; mean age = 46.8; 19% female) explored the impact of a 12 week aerobic exercise program (at least three, 1 hour sessions per week of moderate-intensity aerobic and anaerobic exercise), compared to waitlist control, on self-reported symptoms of PTSD [CAPS; (56)]. There was no indication when intervention took place in relation to experienced trauma. Results revealed that participants who completed the exercise program reported greater reduction in PTSD symptom severity compared to waitlist controls [*F*_(1, 35.3)_ = 4.64, *p* = 0.038]. Further, hyperarousal symptoms were the only cluster that benefitted significantly from the exercise intervention [*F*_(1, 35.2)_ = 4.40, *p* = 0.044]. Additional findings from the same sample suggest that the exercise program was also associated with increased self-reported mindfulness and interoceptive awareness (67).

#### Exercise Plus Standard PTSD Treatment

Two additional studies examined the effect of aerobic exercise training on PTSD symptoms in conjunction with other forms of treatment. A study conducted by Powers et al. (60) compared PTSD symptom changes in community adults with PTSD (*N* = 9; mean age = 34; 89% female) who were assigned to moderate-intensity aerobic exercise (stationary cycling; 70% age predicted heart rate max) prior to 12 prolonged exposure therapy sessions (an evidence-based treatment for PTSD) or to prolonged exposure therapy only (*d* = 2.65, *SE* = 0.92). The results indicated that the group who exercised prior to therapy showed greater improvement in PTSD symptoms relative to the group only participating in therapy.

In another study, inpatients with PTSD (*N* = 81; mean age: 48; 84% male; BMI = 30.1 obese) were assigned to either a 12 week exercise program that included three, 30-min sessions per week of progressive resistance training, walking (daily step count goal = 10,000 steps), and “care as usual” to “care as usual” alone (68). Individuals in the exercise group had significantly greater symptom reduction on a self-report measure of PTSD (PCL-C; *mean difference* = −5.4, 95% CI −10.5 to −0.3, *p* = 0.04), as well as greater reductions on measures of depression, anxiety, stress, weight, percent body fat, and weight circumference compared to the “care as usual” group. The reductions in PTSD symptomatology occurred despite the finding that there were no between-group differences in indicators of cardiorespiratory fitness as measured by the 6-min walk test, resting heart rate, and blood pressure. Pre-to post-intervention within-group differences in cardiorespiratory fitness were not measured. Results from these two studies converge to suggest that exercise combined with other forms of treatment may reduce symptoms above and beyond symptom reduction with standard treatments alone.

The intervention studies to date suggest that exercise may in fact lead to reduction in symptoms among individuals with PTSD. While the existing studies are promising in this regard, many of the studies have small sample sizes. In addition, six of the ten intervention studies lack control groups, and thus do not account for other variables that may contribute to symptom improvement. This is important to note given that in some cases symptoms of PTSD resolve without intervention and only a percentage of the population requires treatment for PTSD symptom resolution. However, half of these studies were conducted in the context of residential treatment programs suggesting the participants were likely constituents that would, in fact, benefit from treatment to promote symptom reduction.

### General Discussion

To summarize, both observational and intervention studies provide support for the notion that aerobic exercise, either alone or in combination with standard treatments, exerts positive mental health benefits among individuals with PTSD. The results are encouraging as positive effects were observed in both civilian and military populations, as well as in both predominately female and male study samples. Based on the pattern of findings across studies and other existing areas of research we briefly review potential mechanisms by which aerobic exercise may exert a positive impact in PTSD symptoms, followed by the limitations of the current studies and potential future directions.

#### Exposure and Desensitization to Internal Arousal Cues

One of the core features of PTSD symptomatology is hyperarousal, which results in sensitivity to physiological arousal cues. Research suggests that repeated exposure to these physiological cues, such as rapid heartbeat, in the context of exercise may increase tolerance of and facilitate desensitization to the physiological sensations (32, 69, 70). However, existing research has focused on this mechanism in anxiety disorders and this effect has been largely unexplored in individuals with PTSD. Nevertheless, the possibility that exercise could result in desensitization to physiological arousal cues in a clinical PTSD sample is consistent with the findings reviewed that suggest that those who engage in vigorous-intensity exercise have fewer hyperarousal symptoms of PTSD (45, 49, 53). In other words, it may be that the vigorous activity, which by definition elicits the greatest increase in physiological arousal cues, is a means of exposure, and reduces the individuals' subjective heightened response to these cues in other (non-exercising) contexts. While we are not aware of quantitative evidence of this exercise-related exposure effect in PTSD populations, a recent case study suggested that this type of exposure might contribute to the therapeutic effects of exercise on PTSD. More specifically, the case study revealed that an individual with PTSD who participated in a 3 month exercise program (2x/week for a total of 90 min) became more aware that his bodily sensations, like pain or fast heartbeat, were not necessarily catastrophic or related to a traumatic event (71). Taken together, it is reasonable to hypothesize that exposure to arousal cues may partly explain the beneficial effect of exercise on PTSD symptomatology.

#### Improved Cognitive Function

A well-replicated finding in the PTSD literature is that of disrupted cognitive control in individuals with PTSD, evidenced by greater distractibility from task irrelevant stimuli and difficulty inhibiting responses to task irrelevant stimuli (72–74). At the center of the cognitive control disturbance is difficulty disengaging attention from salient stimuli that may be interpreted as potentially threatening, even if neutral or positively valenced (75, 76). This impairment in cognitive control may contribute to the maintenance of PTSD symptoms, such as hypervigilance and avoidance (77). In addition to impairments in cognitive control, PTSD is also associated with reduced learning and memory. In particular, individuals with PTSD show reduced encoding of specific episodic details, including autobiographical memories, as well as increased false memory and reduced extinction learning, which also has implications for the maintenance of PTSD symptoms.

To date, we are not aware of any aerobic exercise studies in PTSD that used cognitive outcome measures. Nevertheless, PTSD-related cognitive impairment and the maintenance of PTSD symptoms may be alleviated by improvements in cognition that are often associated with aerobic exercise. To date, the majority of studies on aerobic exercise and cognition have focused on older adults. For instance, extant research among healthy older adults suggests that cardiorespiratory fitness and aerobic exercise positively affect executive function (78, 79) and episodic memory (80–83)—the same cognitive functions negatively impacted by PTSD. Although speculative, it may be the case that aerobic exercise improves executive function and episodic memory function in PTSD, which are deficits that contribute to the core psychiatric symptoms of PTSD, such as re-experiencing, avoidance, and hyperarousal.

#### Alterations in Brain Structure and Function

The presence and severity of PTSD symptoms have been linked to structural gray and white matter abnormalities in the medial temporal lobes and fronto-parietal regions, which underpin executive function and episodic memory. For instance, gray matter changes associated with PTSD include reduced volume in the hippocampus and the anterior cingulate (84–86), reduced volume or cortical thickness in the precuneus, insula, and ventromedial and lateral prefrontal cortex (87, 88). A preliminary quantitative meta-analysis of seven adult onset PTSD studies (89) revealed white matter abnormalities in the cingulum bundle, a white matter tract that links the anterior medial prefrontal cortex to the medial temporal lobes, and the superior longitudinal fasciculus, which connects the frontal and parietal lobes (90). These structural alterations co-occur with functional brain alterations in PTSD, which include alterations in activation and connectivity in amygdala, hippocampal, orbitofrontal cortex and dorsal anterior cingulate cortex (3, 6, 91, 92).

We are not aware of any exercise intervention studies in PTSD that assess brain structure or function. However, there is overlap among the neural correlates of PTSD and the brain regions impacted by aerobic exercise (93), suggesting that exercise-induced brain changes may be a mechanism by which aerobic exercise positively impacts PTSD symptomatology. For instance, aerobic training studies in older adults have revealed increased volume of prefrontal regions, including anterior cingulate cortex, during aerobic training (exercising at up to 70% of their heart rate reserve; HRR) relative to a non-aerobic stretching control group (94). Another study found that increases in physical activity resulting from exercise training were associated with gray matter volume increases in the cingulate gyrus (including both anterior and posterior cingulate cortex), left superior frontal gyrus, left medial parietal cortex, among others (95). After participation in an aerobic exercise program in another study, older adults demonstrated an approximately 2% increase in hippocampal volume, whereas a *decrease* of roughly 1.4% was observed in the stretching control group (96). Increased hippocampal volume may in part be explained by exercise associated increases in neurogenesis in the dentate gyrus subfield of the hippocampus (97, 98), which is negatively impacted in PTSD (84). Cardiorespiratory fitness and aerobic exercise have been positively associated with fronto-parietal and medial temporal lobe white matter tracts including the fornix, cingulum bundle and superior longitudinal fasciculus (99–102). Functional brain alterations have also been associated with aerobic fitness (103–105), and aerobic exercise has been shown to impact activation in many of the same regions implicated in PTSD (93, 106, 107).

#### Normalization of Hypothalamic Pituitary Axis (HPA) Axis Function

The HPA axis is a neuroendocrine system that regulates reactions to stress and physiological processes. In the context of stress, the hypothalamus releases corticotrophin releasing hormone (CRH), which stimulates the pituitary gland to release adrenocorticotropic hormone (ACTH). Ultimately, cortisol is released by the adrenal cortex, which regulates the system by providing negative feedback to the hypothalamus and pituitary gland. Alterations in the HPA axis are a relatively reliable finding in the PTSD literature. More specifically, a recent review of the neuroendocrine characteristics of PTSD indicated that individuals with PTSD tend to express low basal cortisol levels, which is associated with increased levels of CRH, and an exaggerated HPA axis negative feedback loop, while peripheral and central catecholamine levels are increased (108). Further, a review of studies that assess the acute response to laboratory stressors suggests that the HPA axis response among those with PTSD is characterized by an exaggerated cortisol response (109).

Normalization of the HPA axis may be a therapeutic target for PTSD based on its association with PTSD symptom improvement (110, 111). In fact, acute and regular aerobic exercise exert positive effects on the HPA axis, which may promote normalization and reduction in PTSD symptoms. For instance, an early review suggests that acute exercise induces the secretion of ACTH which stimulates the release of cortisol (112). Findings from more recent intervention studies suggest that acute moderate- and vigorous-intensity, but not low-intensity, exercise result in increased levels of cortisol (113, 114), which might target the low basal cortisol levels seen in PTSD populations. Additional research conducted in healthy or other clinical populations provides evidence that acute and regular exercise regulate or improve one's stress-related activation of the HPA axis, including a blunted cortisol response (115–118). The extent to which these findings translate to the PTSD-related HPA axis alterations is unclear because to our knowledge, this effect has only been explored in healthy populations or clinical populations with HPA axis alterations that differ from those observed in PTSD.

#### Activation of the Immune System and Reduced Inflammation

The activation of the HPA axis has pro-inflammatory implications for the immune system (119). While the understanding of the role of inflammation in the etiology and maintenance of PTSD is limited, there is evidence to suggest that PTSD is characterized by increased inflammatory activity. More specifically, a meta-analysis comparing individuals with PTSD and healthy controls, found that PTSD was associated with elevations in pro-inflammatory cytokines such as interleukin-1 beta (IL-1β), interleukin-6 (IL-6), tumor necrosis factor-alpha (TNF-α), interferon-gamma [INF-γ; (120)]. There is also evidence to suggest that there is increased c-reactive protein (CRP), decreased anti-inflammatory markers (e.g., interleukin-8 [IL-8], interleukin-2 [IL-2]) as well as alterations in cell function and genes related to inflammation in populations with PTSD (119, 121–123).

Interestingly, exercise has been shown to influence these same inflammatory pathways in a manner that could possibly counter-act some of the augmentations documented in PTSD. For example, regular aerobic exercise can increase factors involved in immunomodulatory function and diminish pro-inflammatory signaling, through downstream anti-inflammatory effects of acute production of IL-6 (124–127). There is also evidence to suggest that inflammatory alterations might partly explain the effect of exercise on sleep quality (128), which has shown to both moderate and mediate the association between aerobic exercise and PTSD (49, 53). Pro-inflammatory cytokines that are modulated by exercise play a crucial role in sleep regulation, with low concentrations of IL-1 and TNF-α associated with enhanced non-REM sleep (128–130), and high levels linked to sleep disorders. Taken together, the favorable effects of exercise on inflammation and the role of inflammation in sleep regulation support the notion that exercise may be an effective tool for improving PTSD symptoms but requires additional study to directly test this assertion.

#### Limitations

Although the current review of the literature is encouraging, the extant studies have limitations. For instance, many of the studies had small sample sizes, restricted age ranges and/or ethnicities (many predominantly white samples), focused on either females or males, and/or relied on convenience sampling for recruitment. In addition, the populations from these studies are quite variable in terms of severity of PTSD symptoms (ranging from inpatient to trauma-exposed without PTSD), veteran status, and whether they are seeking treatment. While these factors limit the generalizability of each study to dissimilar populations, the positive association between exercise and PTSD across these variable samples provides some evidence for the generalizability of the findings. There are also considerable limitations related to the heterogeneity in research methodology. In terms of measurement, PTSD was assessed using many different self-report measures of PTSD symptoms and only select studies reported on time since traumatic event, which could impact the benefits of exercise on PTSD. There is substantial variability in the exercise measures and protocols administered across studies. First, single-item measures of exercise may hinder the detection of significant findings due to the oversimplification of exercise. Second, many of the studies rely on retrospective self-report measures of exercise, which may be prone to bias, inaccuracies, and misunderstanding of questions [e.g., (50–52)], rather than objective measures of exercise or fitness levels. In addition, the majority of the existing intervention studies failed to implement rigorous pre- and post- intervention fitness assessments and few included a control group. Therefore, some of these early studies are not able to demonstrate that alterations in PTSD were specific to alterations in fitness (as opposed to common factors). As more randomized clinical trials of exercise and PTSD appear in the literature, it will be possible to complete a quantitative meta-analysis of the effects of aerobic exercise on PTSD symptoms. However, given the heterogeneity in the study methods outlined above, and in particular the lack of an adequate number of studies that included a control group, we felt a quantitative analysis of the literature was not appropriate at this time. Nevertheless, these early and promising findings as well as the consideration of some of the potential mechanisms by which exercise could exert a positive impact in PTSD provide motivation for further investigation.

#### Future Directions

While there is evidence that aerobic exercise may positively impact PTSD symptoms, appropriately powered randomized clinical trials are required to more firmly establish the relationship between exercise and PTSD. At a minimum, aerobic exercise interventions should adhere to CDC's recommended guidelines for cardiovascular fitness-improving activity (i.e., 150 min of moderate-intensity or 75 min of vigorous-intensity exercise per week). Future studies should implement gold standard pre- and post-intervention diagnostic interviews to determine the presence and severity of PTSD symptomatology, such as the Clinician-Administered PTSD Scale for DSM-5 (CAPS-5) and a progressive, graded exercise test to assess cardiorespiratory fitness. Objective assessment of exercise adherence could be assessed through the use of accelerometers paired with chest-strap heart rate monitors (shown to be superior to wrist-based heart rate monitors), which would allow researchers to objectively assess the frequency, intensity, and time (duration) of exercise during the intervention. Moreover, use of accelerometers would also provide insight about non-exercise related physical activity, such as occupational, domestic, or travel-related physical activity, which could potentially moderate treatment effects. Given the constellation of psychiatric, cognitive, neural, and general health markers of PTSD, it is also important to consider the use of cognitive, neuroimaging, and neurobiological outcome measures. These data may elucidate the mechanisms of exercise-induced symptom reduction in PTSD, and once clarified, may provide insight as to the dosage of exercise required to attenuate PTSD symptoms. Moreover, given the current state of the literature, we have focused exclusively on aerobic fitness. Other exercise interventions, which have yet to be thoroughly explored, including resistance training or combined aerobic and resistance training, could also elicit significant reductions in PTSD symptoms. Further research on exercise and PTSD using some of these suggestions would help to better understand the effects of exercise on PTSD and might shed light on the possible mechanisms by which exercise improves PTSD and its sequelae.

#### Conclusion

Aerobic exercise is a low-cost, widely accessible activity known to provide multiple health benefits, including cardiovascular health and musculoskeletal health, as well as reduced rates of co-morbidity and mortality (28). Moreover, aerobic exercise is not associated with the stigma of standard mental health treatment (19). There is growing evidence of the beneficial effects of exercise on mental health disorders, including depression and anxiety (32, 33, 35, 36). Our review of the literature suggests aerobic exercise may also reduce PTSD symptomatology across a variety of populations providing evidence for the clinical utility of exercise as a form of treatment. Additional RCTs are required to provide more definitive evidence of a causal relationship between exercise and PTSD. Nevertheless, we are not aware of any negative effects of exercise on PTSD symptoms. Thus, in the absence of medical contraindications for exercise, providers for patients with PTSD may consider prescribing aerobic exercise. To date, the precise mechanisms by which the beneficial effects of exercise may influence PTSD are unknown. However, there are multiple psychological, cognitive, and neurobiological routes that may underpin exercise-induced improvements in PTSD. Methodologically-rigorous research is required to provide more definitive evidence of a positive effect of aerobic exercise on PTSD and identify the mechanisms of exercise-induced alterations in PTSD.

## Author Contributions

SH generated the idea and outline for the project. NH completed the literature search and wrote the review, with JH and SH contributing conceptual guidance and writing.

### Conflict of Interest Statement

The authors declare that the research was conducted in the absence of any commercial or financial relationships that could be construed as a potential conflict of interest.
